# Health Insurance and Neighborhood Deprivation as Determinants of Diagnostic Delays and Survival in Breast Cancer

**DOI:** 10.3390/healthcare14050676

**Published:** 2026-03-07

**Authors:** Axel Gierbolini-Bermúdez, Maira A. Castañeda-Avila, Marjorie Vázquez-Roldán, Tonatiuh Suárez-Ramos, Carlos R. Torres-Cintrón, Rosa Román-Oyola, Karen J. Ortiz-Ortiz

**Affiliations:** 1 Division of Cancer Control and Population Sciences, Comprehensive Cancer Center, University of Puerto Rico, San Juan 00935, Puerto Rico; mavazquez@cccupr.org (M.V.-R.); kareno@cccupr.org (K.J.O.-O.); 2 Puerto Rico Central Cancer Registry, Comprehensive Cancer Center, University of Puerto Rico, San Juan 00935, Puerto Rico; tonatiuhs@cccupr.org (T.S.-R.); carlost@cccupr.org (C.R.T.-C.); 3 Department of Population and Quantitative Health Sciences, University of Massachusetts Chan Medical School, Worcester, MA 01605, USA; maira.castanedaavila@umassmed.edu; 4 Occupational Therapy Program, School of Health Professions, Medical Sciences Campus, University of Puerto Rico, San Juan 00936, Puerto Rico; rosa.roman2@upr.edu; 5 Department of Health Services Administration, Graduate School of Public Health, Medical Sciences Campus, University of Puerto Rico, San Juan 00936, Puerto Rico

**Keywords:** breast cancer, delay to diagnosis, health insurance, neighborhood deprivation, social determinants of health

## Abstract

**Background/Objectives**: Breast cancer (BC) represents a major public health problem that is influenced by social and systemic factors. This study evaluates disparities in the BC care continuum based on health insurance type and determines whether these patterns differ according to neighborhood-level deprivation. **Methods**: Using the Puerto Rico Central Cancer Registry-Health Insurance Linkage Database, we conducted a retrospective cohort study of women aged ≥18 years and diagnosed with BC in Puerto Rico between 2012 and 2016. The main outcomes were diagnostic delay (>60 days) and six-year mortality. Insurance type (private, Medicare, Medicaid, and dual enrollment in Medicare and Medicaid) was the main predictor, with neighborhood deprivation as a modifier. Logistic and Cox models assessed delay and survival, adjusting for key covariates. **Results**: Disparities in diagnostic delays and risk of death across insurance types were most evident in areas with low to average deprivation, whereas, in neighborhoods with above-average to highest deprivation, these differences diminished for diagnostic delay and disappeared for risk of death. **Conclusions**: These findings reveal that neighborhood environment, an intermediary social determinant of health, may affect the timeliness and quality of care provided to women diagnosed with BC.

## 1. Introduction

Breast cancer (BC) poses a major public health concern, ranking as the fourth leading cause of death and the most frequently diagnosed cancer among women in the United States [1]. According to the Puerto Rico Central Cancer Registry (PRCCR), BC was the most diagnosed cancer among women in Puerto Rico from 2018 to 2022, accounting for 31.4% of cases and 17.7% of cancer-related deaths [2]. The cancer care continuum (CCC), as explained by Taplin et al. in their model, encompasses the full range of services provided to individuals, including prevention, detection, diagnosis, treatment, and survivorship care, and may involve multiple stakeholders such as healthcare providers, health systems, and communities [3]. Failures at any stage of the CCC or during transitions between stages can compromise quality of care. For example, in BC, delays between detection and diagnosis are associated with late-stage diagnosis [4]. Late-stage diagnosis has been associated with an increased risk of mortality; thus, avoiding diagnostic delays is crucial [5].

The CCC model, according to Taplin et al., also highlights the role of the social context, or social determinants of health (SDH), in shaping patient experience [3]. The model of Solar et al. groups SDH in two categories: *structural SDH*, such as social, economic, and political policies, which are difficult to measure directly; and *intermediary SDH*, which include social support, healthcare system, and community factors (housing, job, infrastructure) [6]. Barriers within these domains can produce avoidable disparities in outcomes and quality of life [3]. Evidence from the United States mainland and Puerto Rico indicates that efforts to reduce these disparities often fall short when broader systemic and community-level factors are not addressed [7,8]. For example, health insurance type (e.g., private, Medicare, or Medicaid) modifies patient interactions with the healthcare system. Prior research on different cancer sites (e.g., BC, colorectal cancer, and non-small cell lung cancer) in Puerto Rico has shown poorer cancer outcomes among Medicaid recipients compared with privately insured patients [9,10,11,12,13]. However, because SDH are complex and shaped by healthcare structures, further research is needed to identify other factors, beyond health insurance, that contribute to these disparities in health outcomes. One way to do this is to measure intermediary determinants and their associations with health outcomes.

Neighborhood-level conditions, such as the intermediary determinant of socioeconomic deprivation, may also influence disparities in diagnosis and survival [14,15]. Studies have found variation in cancer incidence and mortality across Puerto Rican municipalities by socioeconomic status [16]. For screen-detectable cancers, including breast, colorectal, and prostate, one study observed higher incidence rates in more affluent municipalities [16]. However, there was no difference in mortality rates, underscoring the importance of localized analyses (e.g., sub-county or census tract levels) that better capture neighborhood conditions. The lack of such data in low-income settings, such as Puerto Rico, has impeded research on neighborhood-level influences [2,17,18]. Studies have found that health insurance type (e.g., private, Medicare, or Medicaid) modifies patient interactions with the healthcare system. For example, evidence from a study in the state of Georgia shows that neighborhood-level conditions modify how health insurance status influences treatment delays in women with BC: insurance has less of an impact in highly deprived neighborhoods [19]. However, a key gap in the literature is whether neighborhood deprivation modifies the relationship between diagnostic delays and mortality, particularly in Puerto Rico, where socioeconomic conditions differ markedly from those on the United States mainland. This study addresses that gap by examining these interactions using census-tract–level data, providing new insight into how systemic and neighborhood factors shape disparities in a predominantly Hispanic population.

This study seeks to expand the information available on BC among women by simultaneously considering systemic and neighborhood-level SDH, with a localized geographic focus. The purpose is to assess disparities along the CCC based on health insurance type and to determine whether these disparities differ according to neighborhood-level deprivation among women diagnosed with BC in Puerto Rico. Specific aims were to evaluate whether neighborhood-level deprivation modifies the association between: (1) health insurance plan and delay in cancer diagnosis and (2) health insurance plan and risk of death among women diagnosed with BC in Puerto Rico between 2012 and 2016.

## 2. Materials and Methods

### 2.1. Study Design and Data Source

This retrospective cohort study analyzed data from the Puerto Rico Central Cancer Registry-Health Insurance Linkage Database (PRCCR-HILD), which includes patient data, clinical tumor information, pathological data, and claims information for more than 85% of all cancer cases reported in Puerto Rico. Claims information includes data from Medicaid, Medicare, and private insurance. PRCCR-HILD includes all available claims for the patient both before and after diagnosis, allowing us to potentially study pre-diagnosis, diagnosis, treatment, and post-treatment events. Data were analyzed using Stata version 18.5 BE (StataCorp LP, College Station, TX, USA).

### 2.2. Cohort Selection

This study focused on women aged 18 years or older who were diagnosed with BC (confirmed by pathology) in Puerto Rico between 2012 and 2016. We evaluated the mammography-to-biopsy clinical pathway, which is the most common diagnostic route for BC. This enhanced methodological rigor by creating a more homogeneous cohort. We ensured that participants’ records included date of diagnosis and complete information on health insurance enrollment one year before and after diagnosis. Exclusion criteria included participants without assigned deprivation index data at the census tract level, those from the Department of Veterans Affairs, and those with inflammatory BC, due to differences in diagnosis and treatment protocols. Cases that were detected and diagnosed on the same day were omitted because these patients are considered to have only a diagnosis phase, without a detection phase. Finally, we excluded individuals with missing data on marital status, diagnosis confirmation, stage at diagnosis, or comorbidities.

### 2.3. Study Variables

#### 2.3.1. Primary Outcomes

The outcomes of interest were (1) delay in the transition from detection to diagnosis and (2) the risk of death over a six-year follow-up. The delay from detection to diagnosis was calculated as the interval between the last mammogram (including 2D and 3D mammography) and the pathological diagnosis. Other supplemental detection methods, like sonograms or MRIs, were not included. The delay was categorized according to a 60-day threshold (>60 days or ≤60 days), based on previous literature [4,19] and the standards of the U.S. Centers for Disease Control and Prevention’s National Breast and Cervical Cancer Early Detection Program [20]. The PRCCR database contains patient information at death, as recorded in the death certificate issued by the Puerto Rico Demographic Registry. Survival time was calculated from the date of diagnosis with a six-year follow-up.

#### 2.3.2. Primary Predictor

The primary predictor variable for this study was the type of health insurance at the moment of diagnosis. This variable was classified as private, Medicare, Medicaid, and dual (enrollment in both Medicare and Medicaid).

#### 2.3.3. Modifier Variable

The modifier variable in this study was the patient’s neighborhood-level deprivation. This was based on a previous study that identified an interaction between health insurance and deprivation [19]. To measure neighborhood-level deprivation, we used an adaptation by Diez et al. of the U.S. Census Bureau’s deprivation index at the census tract level [21,22,23]. The deprivation index is calculated based on 17 indicators across four domains: poverty, educational level, housing, and employment. This index was originally divided into five quintiles: lowest, below-average, average, above-average, and highest, representing increasing levels of deprivation. Based on procedures from previous studies [24,25,26], we collapsed these quintiles into two categories: **lowest-to-average deprivation**, which comprised the lowest three quintiles (lowest, below-average, and average), and **above-average-to-highest deprivation**, which comprised the top two quintiles (above-average and highest). Then, we stratified by the two resultant categories (**lowest-to-average deprivation** and **above-average-to-highest deprivation**). This decision was made to ensure an adequate sample size within each category, which aligns with prior literature indicating meaningful differences primarily between lower and higher deprivation contexts [19,24,25,26]. Collapsing categories also reduced dispersion and improved model stability while preserving the contrast most relevant to assessing disparities. To validate this choice, we assessed interactions between the deprivation index and other variables in a Cox proportional hazards model using a likelihood ratio test.

#### 2.3.4. Independent Variables

Diagnostic delay and risk of death were adjusted by characteristics at the time of diagnosis, including health insurance, deprivation index, age groups, marital status, comorbidity index, and stage at diagnosis. Age at diagnosis was classified as less than 50, 50 to 69, and 70 years or older. Marital status was categorized as married and unmarried. For the comorbidity index, we used the National Cancer Institute-modified Charlson’s comorbidity index, which considers 17 comorbidities and is calculated based on their quantity and severity [27]. For stage at diagnosis, we used the SEER Summary Stage 2018 from the Surveillance, Epidemiology, and End Results (SEER) Program [28]. The summary stage at diagnosis describes how far cancer has spread from its origin, and for this study, it was categorized as localized, regional, or distant. Stage at diagnosis was treated as an independent prognostic factor in the survival analysis due to its known impact on mortality risk. Since diagnostic delay occurs before diagnosis and can influence the stage, and because the stage directly affects survival outcomes, we did not include stage in the delay model to prevent it from being considered a predictor of delay. Instead, stage was incorporated only in the survival model to accurately reflect its influence on mortality.

### 2.4. Statistical Analysis

#### 2.4.1. Delay from Detection to Diagnosis

Logistic Regression: A logistic regression model was used to evaluate the strength and direction of the association between patient health insurance and diagnostic delay, stratified by deprivation index. In addition, to evaluate the association between neighborhood deprivation and diagnostic delay, a supplementary analysis was performed without this stratification and without collapsing the deprivation index quintiles. Both adjusted models included marital status, age group, and comorbidity index. Results are presented as adjusted odds ratios (aOR) with 95% confidence intervals and *p*-values.

#### 2.4.2. Survival and Risk of Death

Survival Analysis: We evaluated six-year survival rates by health insurance type, stratifying by deprivation index. We estimated survival curves using the Kaplan–Meier method. The log-rank test was used to describe the differences between curves.

Risk of Death Analysis: Cox regression analysis was performed to assess whether neighborhood deprivation modifies the association between insurance type and risk of death over a 6-year follow-up period. In the adjusted models, we included age group, comorbidity index, marital status, and stage at diagnosis. Results are presented as adjusted hazard ratios (aHR) with 95% confidence intervals and *p*-values.

## 3. Results

**Figure 1:**
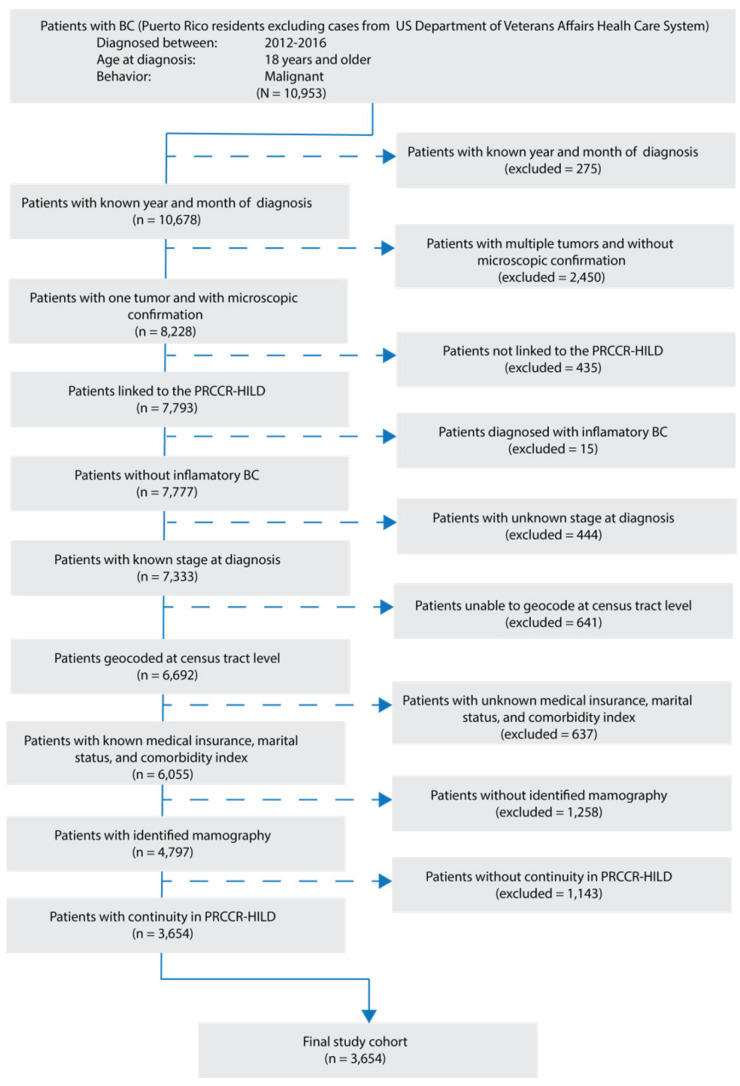
Flowchart diagram of cohort selection.

After applying the selection criteria, the cohort included 3654 women (Figure 1). Most patients were diagnosed between the ages of 50 and 69 years (57.25% of the cohort), followed by patients 70 years or older (31.83%) (Table 1). Those living in neighborhoods with the **lowest to average deprivation** represented 58.10% of participants: 13.77% of the overall cohort resided in neighborhoods categorized as lowest deprivation and 23.29% in neighborhoods categorized as below-average. Those in neighborhoods with **above-average to highest deprivation** represented 41.90% of participants: 23.67% of the overall cohort resided in neighborhoods categorized as above-average deprivation and 18.23% in the highest deprivation neighborhoods. In terms of insurance type, 31.96% had Medicare, 25.92% had Medicaid and 26.16% had private insurance. Most patients were diagnosed at a localized stage (65.98%), followed by those diagnosed at a regional stage (30.87%). Most patients had a comorbidity index of zero (64.53%), and 51.53% were married.

### 3.1. Delay from Detection to Diagnosis

**Figure A1:**
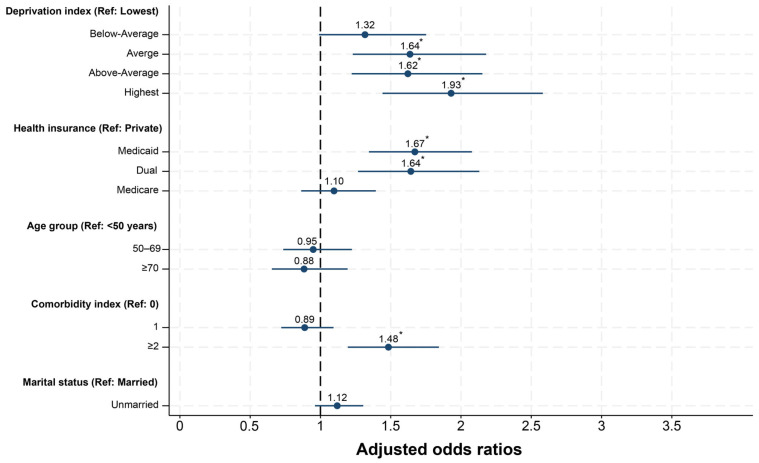
Association between delay in diagnosis and patients’ characteristics. Adjusted odds ratios were estimated for BC patients. 95% confidence intervals were represented by solid lines. *p*-values < 0.05 were considered statistically significant and indicated by *.

Approximately one-quarter of the population experienced a delay between detection and diagnosis of over 60 days. In our analysis of diagnostic delay by deprivation quintile, the logistic regression model showed that patients living in neighborhoods with average and above-average deprivation were 64% (aOR: 1.64; 95% CI: 1.23–2.18) and 62% (aOR: 1.62; 95% CI: 1.22–2.15) more likely to experience diagnostic delay, respectively, than patients living in neighborhoods with the lowest deprivation. Furthermore, patients living in the highest deprivation neighborhoods have almost twice the delay (aOR: 1.93; 95% CI: 1.45–2.58) as those living in neighborhoods with the lowest deprivation (Figure A1).

**Figure 2:**
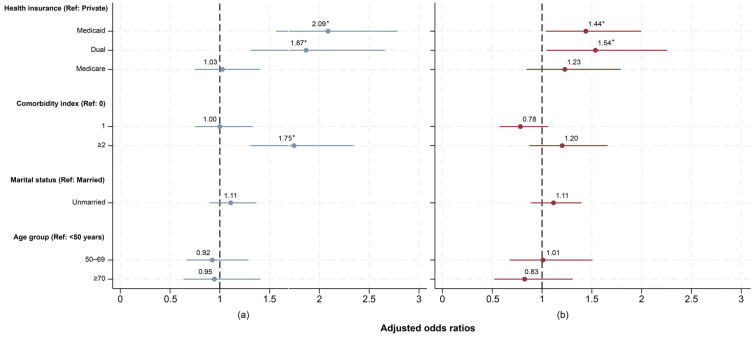
Association between delay in diagnosis and patients’ characteristics by deprivation index. Adjusted ORs were estimated for BC patients in (**a**) **lowest to average deprivation**, and (**b**) **above-average to highest deprivation**. 95% confidence intervals were represented by solid lines. *p*-values < 0.05 were considered statistically significant and indicated by *.

The results of the logistic regression model showed that neighborhood deprivation modifies the relationship between health insurance and delays in diagnosis. We found that in the **lowest to average deprivation** neighborhoods, patients with dual insurance were 87% more likely to experience a delay than those with private insurance (aOR: 1.87; 95% CI: 1.31–2.66), and Medicaid patients were more than twice as likely to experience delays (aOR: 2.09; 95% CI: 1.56–2.78) (Figure 2a).

In **above-average to highest deprivation** neighborhoods, there were also significant differences in diagnostic delay for patients with either Medicaid or dual insurance compared with those with private insurance (Figure 2b). Patients with Medicaid were 44% more likely (aOR: 1.44; 95% CI: 1.04–2.00), and those with dual insurance were 54% more likely (aOR: 1.54; 95% CI: 1.05–2.26), to experience a delay in diagnosis than patients with private insurance.

To further explore these unexpected patterns, we performed a cross-tabulation between health insurance and delay status (delayed vs. not delayed), stratified by deprivation index (Table 2). We found that patients with private insurance in **above-average to highest deprivation** neighborhoods were 7% more likely to experience a delay than those with private insurance in **lowest to average deprivation** neighborhoods. Likewise, those with Medicare in **above-average to highest deprivation** neighborhoods were 8% more likely to experience a delay than their counterparts in **lowest to average deprivation** neighborhoods. However, in patients with Medicaid and dual insurance, the differences were 1% and 0%, respectively.

### 3.2. Survival and Risk of Death

#### 3.2.1. Survival

**Figure 3:**
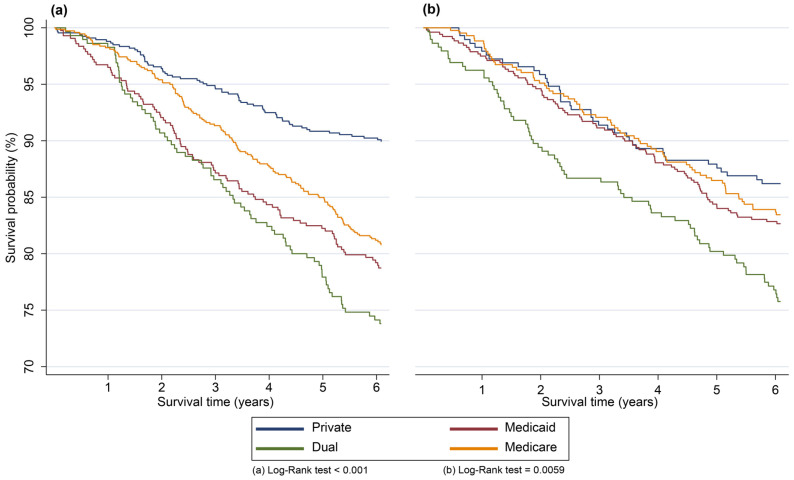
Kaplan–Meier survival curves among BC patients. Kaplan–Meier survival curves were estimated up to 6 years for BC patients in neighborhoods with (**a**) **lowest to average deprivation** and (**b**) **above-average to highest deprivation**. In each analysis, survival curves were compared across four different health insurance plans. Statistical significance for the log-rank test was defined as a *p*-value < 0.05, and the test was used to compare survival curves among health insurance plans.

A six-year survival analysis was conducted to describe the outcomes by health insurance type and the mediating effect of the deprivation index (**lowest to average deprivation** vs. **above-average to highest deprivation**) (Figure 3). Among patients with the **lowest to average deprivation**, those with Medicaid, dual insurance, and Medicare had lower six-year survival probabilities (79.21%, 74.14%, and 81.19%, respectively) compared to those with private insurance (90.24%) (Log-Rank Test *p*-value < 0.05). Similar trends were observed in two- and four-year survival probabilities (Table 3). In contrast, among patients in the **above-average to highest deprivation** neighborhood, those with private, Medicare, and Medicaid insurance had similar survival probabilities at six years (86.21%, 83.92%, and 82.85%, respectively), while patients with dual insurance showed a lower survival probability of 76.8% (Log-Rank Test *p*-value < 0.05). Notably, among patients residing in areas with the **lowest to average deprivation** and with **above-average to highest deprivation**, there was approximately a 7% difference in survival probability at six years between those covered by age-based insurance programs (Medicare and dual insurance). In contrast, patients with private insurance in areas with the **lowest to average deprivation** had 11% higher survival probability at six years compared to those with Medicaid. When comparing patients with private insurance and Medicaid in **above-average to highest deprivation** areas, the difference decreased from 11% to 3.36%.

#### 3.2.2. Risk of Death

**Figure 4:**
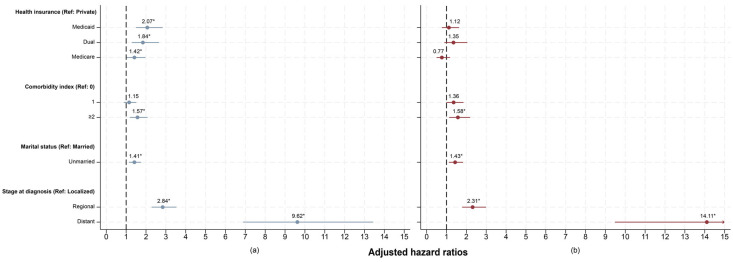
Magnitude of the association between risk of death and different characteristics among BC patients. Adjusted hazard ratios (aHRs) were estimated for BC patients in neighborhoods with (**a**) **lowest to average deprivation**, and (**b**) **above-average to highest deprivation**. 95% confidence intervals were represented by solid lines. *p*-values < 0.05 were considered statistically significant and indicated by *. aHRs were stratified by age group and adjusted by health insurance, comorbidity index, marital status, and stage at diagnosis. The upper confidence interval for distant stage at diagnosis in panel b was truncated from the graph because it was substantially higher than the other estimates (aHR: 14.11; 95% CI: 9.48, 20.99). The arrow indicates this truncated confidence interval.

The results of the adjusted Cox model show that neighborhood deprivation modifies the statistical relationship between type of health insurance and the risk of death (Figure 4). Because the proportional hazards assumption was violated (*p*-value < 0.05), we stratified the model by age group. Age was selected as the stratification variable because it was the primary source of non-proportionality in residual diagnostics, which allowed us to satisfy the assumption while preserving the interpretation of the main exposure variables [26]. Additionally, within each Cox model (lowest to average deprivation and above-average to highest deprivation), no significant interaction terms were observed between health insurance and the other independent variables (*p*-value > 0.05). We found that the patients from the **lowest to average deprivation** neighborhoods with Medicaid insurance had over twice the risk of death compared to those with private insurance (aHR: 2.07; 95% CI: 1.50–2.84). Patients with dual insurance had an 84% higher risk of death (aHR: 1.84; 95% CI: 1.28–2.65), and those with Medicare had a 42% higher risk (aHR: 1.42; 95% CI: 1.02–1.97) than privately insured patients, respectively (Figure 4b).

Among patients living in **above-average to highest deprivation** neighborhoods, the difference in health insurance was not significant. Patients insured with Medicaid had 12% (aHR: 1.12; 95% CI: 0.77–1.64) and those with dual insurance had 35% (aHR: 1.35; 95% CI: 0.89–2.05) higher risk of death compared to those with private insurance, respectively. Meanwhile, patients insured with Medicare had a 23% lower risk of death (aHR: 0.77; 95% CI: 0.50–1.18) when compared with those with private insurance (Figure 4b).

## 4. Discussion

This study evaluates whether the neighborhood deprivation index of BC patients in Puerto Rico modifies the relationship between: (1) health insurance and delays in diagnosis and (2) health insurance and risk of death. Previous studies on the island on various cancers (e.g., BC, colorectal cancer, and non-small cell lung cancer) have shown poorer cancer outcomes among Medicaid recipients compared with privately insured patients [9,10,11,12,13]. Our findings generally support this pattern; however, the magnitude of observed differences varied by neighborhood deprivation level. Specifically, disparities in diagnostic delays across insurance types were most evident in areas with low to average deprivation, whereas, unexpectedly, in neighborhoods with **above-average to highest deprivation**, these differences diminished or disappeared. The apparent attenuation of disparities in **above-average to highest deprivation** neighborhoods may reflect an increase in delays among privately insured patients, rather than improvements among Medicaid or dual-insured patients. Based on a subsequent cross-tabulation analysis, we argue that the apparent attenuation is likely due to the observed increase in delay among patients with private insurance (our reference level) residing in **above-average to highest deprivation** neighborhoods, compared to their counterparts in **lowest to average deprivation** neighborhoods. This aligns with findings from Arwan et al.’s study [19]. Although they measured delay from diagnosis to treatment (while we evaluated delay from detection to diagnosis), they reported that in low-deprivation neighborhoods, privately insured women were significantly less likely to experience treatment delays than other insurance groups [19]. In contrast, in high-deprivation areas, patients from different insurance groups (including private) were equally likely to experience delays [19]. However, further studies are needed to better understand the relationship between health insurance, high deprivation index, and delays in diagnosis. Furthermore, Roche et al. found that neighborhoods with the highest deprivation and greater concentrations of racial minorities exhibited higher proportions of women diagnosed at distant stages, underscoring the influence of socioeconomic context on cancer outcomes [29].

We also found that neighborhood deprivation modifies the relationship between health insurance and the risk of death. In the **lowest to average deprivation** neighborhoods, individuals with different insurance types (private, Medicare, Medicaid, and dual) appear to differ substantially in underlying characteristics, whereas in **above-average to highest deprivation** neighborhoods, these differences are no longer significant.

These findings must be interpreted with caution. The observed reduction in risk of death should not be interpreted as neighborhood deprivation acting as some kind of protective factor. Rather, although the insurance types were the same in both deprivation groups, the data suggest that insurance advantages, such as those typically associated with private coverage, may appear less pronounced in highly deprived areas. Consistent with patterns observed for diagnostic delays, we hypothesized that patients with private insurance living in highly deprived neighborhoods would have a higher observed risk of death than their counterparts in less deprived areas. This increase brings their outcomes closer to those of Medicaid enrollees.

Although a previous study in Puerto Rico by M. Torres-Cintrón et al. reported no significant differences in mortality rates across municipalities, our findings indicate that social conditions in the CCC may interact with individual-level factors and are likely associated with patient outcomes [16]. This interaction may reflect the use of more granular geographic units in the present study, such as census tracts, which better capture neighborhood socioeconomic conditions. These results suggest that survival disparities may be masked when analyses are conducted at broader geographic levels.

Our analysis strategies enabled the integration of census tract data to enhance analysis of the continuum of healthcare for women diagnosed with BC. We must acknowledge various limitations in our study. The extent of generalizability of the results is limited to the scope of the PRCCR-HILD, regardless of geo-codification. Although the PRCCR-HILD dataset captures more than 85% of all cancer cases reported in Puerto Rico and provides a highly representative source of breast cancer data, not all patients had continuous insurance enrollment. This may limit the completeness of claims information and introduce potential selection bias, which should be considered when interpreting our findings. Another limitation is the temporality of the variables, due to the deprivation index and the health insurance plan collected at the time of diagnosis; these variables are not necessarily constant over time, and changes before or after the diagnosis can affect health outcomes. Future studies could incorporate other methodologies that evaluate this dynamic, for example, using dominant health insurance versus using an index date. Another limitation was that diagnostic delay was defined only within the transition of mammography followed by biopsy; events detected through other procedures or occurring outside this sequence were not addressed, which may lead to misclassification and does not fully reflect all diagnostic transitions. In addition, we want to acknowledge the potential influence of unmeasured confounders such as individual socioeconomic status, access to healthcare facilities, provider characteristics, prior screening history, and tumor subtype (ER/PR/HER2) that may have impacted the observed associations. Regarding tumor subtypes, since 21% of the patients in the study had an unknown status for this variable, we performed a sensitivity analysis. The results showed that the hazard ratios did not differ substantially, reinforcing our decision not to include this confounder. Future studies could explore these confounders and their effect on the deprivation index. During the six-year follow-up period, Puerto Rico also experienced several significant environmental events. In 2017, the island was affected by two major hurricanes, Irma and Maria, which disrupted all infrastructure, compromised the health system, and influenced mortality rates. To address this potential bias, a sensitivity analysis was conducted by excluding patients who died within three months following hurricanes Irma and Maria. This exclusion did not substantially alter the hazard ratios; therefore, we included those cases in our analysis.

## 5. Conclusions

Our findings support the notion that intermediary social determinants of health, as reflected in neighborhood environment, may affect the timeliness and quality of care provided to women diagnosed with breast cancer. Systemic factors such as insurance type and community-level deprivation are associated with differences in how patients move through each phase from detection to diagnosis and ultimately survival. The disparities observed suggest that private insurance and favorable social conditions are associated with more favorable outcomes, particularly in less deprived areas where healthcare resources are more abundant and competitive. Efforts to improve cancer outcomes in Puerto Rico must address both systemic and community-level barriers. Policy interventions should consider the interplay between insurance structures and localized socioeconomic conditions. Future research should continue to employ multilevel approaches and incorporate granular geographic data to refine our understanding of these disparities and inform targeted strategies for equitable cancer care delivery.

## Figures and Tables

**Table 1:** Demographic and clinical characteristics of patients with BC.

Characteristics	Count	Percentage (%)
**Age at diagnosis**		
<50	399	10.92
50–69	2092	57.25
≥70	1163	31.83
**Deprivation index**		
*Lowest to average*	*2123*	*58.10*
Lowest	503	13.77
Below-average	851	23.29
Average	769	21.05
*Above-average to highest*	*1531*	*41.90*
Above-average	865	23.67
Highest	666	18.23
**Health insurance**		
Private	956	26.16
Medicare	1168	31.96
Medicaid	947	25.92
Dual	583	15.96
**Stage at diagnosis**		
Localized	2411	65.98
Regional	1128	30.87
Distant	115	3.15
**Comorbidity index**		
0	2358	64.53
1	705	19.29
≥2	591	16.17
**Marital status**		
Married	1883	51.53
Unmarried	1771	48.47
**Delay in diagnosis**		
>60 days	946	25.89
≤60 days	2708	74.11
**TOTAL**	**3654**	**100.00**

**Table 2:** Crosstabulation between health insurance and delay status stratified by deprivation index.

	Low to Average Deprivation				Above-Average to Highest Deprivation				Change
	No Delay		Delay		No Delay		Delay		
	Count	%	Count	%	Count	%	Count	%	%
Private	549	82%	117	18%	218	75%	72	25%	7%
Medicare	592	80%	147	20%	310	72%	119	28%	8%
Dual	196	68%	94	32%	197	67%	96	33%	0%
Medicaid	295	69%	133	31%	351	68%	168	32%	1%

**Table 3:** Survival estimates for 2, 4, and 6 years among BC patients in Puerto Rico, 2012–2016.

Deprivation Index	Health Insurance	Survival Estimates (%)		
		2 Years	4 Years	6 Years
Lowest to average	Dual	90.69	82.41	74.14
	Medicaid	92.06	84.35	79.21
	Medicare	95.40	87.69	81.19
	Private	96.40	92.49	90.24
Above-average to highest	Dual	89.42	83.62	76.79
	Medicaid	94.61	88.05	82.85
	Medicare	95.10	89.04	83.92
	Private	95.86	89.31	86.21

## Data Availability

The data that supports this study’s findings comes from the PRCCR database. Due to the confidentiality agreement between PRCCR and the authors, the clinical data from this study is not publicly available. However, investigators can obtain this data through PRCCR following the confidentiality procedures and requesting it through the following site: https://rcpr.org/Datos-de-C%C3%A1ncer/Acceso-a-Datos (accessed on 27 November 2025).

## Abbreviations

The following abbreviations are used in this manuscript:

BC	Breast Cancer
PRCCR	Puerto Rico Central Cancer Registry
CCC	Cancer Care Continuum
SDH	Social Determinants of Health
PRCCR-HILD	Puerto Rico Central Cancer Registry-Health Insurance Linkage Database
CDC	The Centers for Disease Control and Prevention
aOR	Adjusted odds ratios
aHR	Adjusted hazard ratios
